# Early Alterations of Lymphocyte Subsets in Acute Respiratory Distress Syndrome Caused by *Acinetobacter baumannii* Pneumonia: A Prospective Observational Study

**DOI:** 10.3389/fmed.2021.762724

**Published:** 2021-10-11

**Authors:** Wei Cheng, Jiahui Zhang, Dongkai Li, Guangxu Bai, Wen Han, Jianwei Chen, Hao Wang, Na Cui

**Affiliations:** ^1^State Key Laboratory of Complex Severe and Rare Diseases, Department of Critical Care Medicine, Peking Union Medical College Hospital, Chinese Academy of Medical Science and Peking Union Medical College, Beijing, China; ^2^Department of Critical Care Medicine, Beijing Jishuitan Hospital, Beijing, China

**Keywords:** Acinetobacter *baumannii*, lymphocyte subset counts, acute respiratory distress syndrome, prognosis, ARDS

## Abstract

**Background:** To prospectively observe the early alterations of lymphocyte subsets in ARDS caused by Acinetobacter *baumannii*.

**Methods:** ARDS patients admitted to our ICU between January 1, 2017 and May 30, 2020 were selected. We enrolled all the pulmonary ARDS caused by Acinetobacter *baumannii* pneumonia who required mechanical ventilation or vasopressors. All the available clinical data, follow up information and lymphocyte subsets were recorded.

**Results:** Eighty-seven of all the 576 ARDS patients were enrolled. The 28-day mortality of the enrolled patients was 20.7% (18/87). The T lymphocyte count (452 vs. 729 cells/ul, *P* = 0.004), especially the CD8^+^ T lymphocyte count (104 vs. 253 cells/ul, *P* = 0.002) was significantly lower in non-survivors, as were counts of the activated T cell subsets (CD8^+^CD28^+^ and CD8^+^CD38^+^). The CD8^+^ T cell count was an independent risk factor for 28-day mortality, and a cutoff value of 123 cells/ul was a good indicator to predict the prognosis of ARDS caused by Acinetobacter *baumannii* pneumonia, with sensitivity of 74.6% and specificity of 83.3% (AUC 0.812, *P* < 0.0001).

**Conclusions:** Lower CD8^+^ T cell count was associated with higher severity and early mortality in ARDS patients caused by Acinetobacter *baumannii* pneumonia, which could be valuable for outcome prediction.

## Background

*Acinetobacter baumannii (A. baumannii)* is an opportunistic pathogen and one of the most common causes of hospital-acquired pneumonia, resulting from the increasingly serious occurrence of antibiotic resistance (1). Acute respiratory distress syndrome (ARDS) caused by *A. baumannii* pneumonia has significantly high mortality (2). It follows that there has been growing interest in identifying biological sub-phenotypes of ARDS patients (3). Measuring plasma biomarkers in ARDS can help find subgroups of patients those share important host-response features and/or those have worse clinical outcomes. Li et al. (4) found that neutrophil to lymphocyte ratio (NLR) was significantly associated with 28-day mortality in patients with ARDS, and NLR was related to the severity of ARDS. However, how the lymphocyte counts changed in NLR and their correlation with prognosis were not clearly illustrated.

Traditionally, *A. baumannii* was thought to cause extracellular infection, and innate immunity played a vital role in the defense against *A. baumannii* infection. Monocytes release tumor necrosis factor (TNF) to recruit granulocytes, which phagocytize bacteria or produce reactive oxygen species. Immature dendritic cells (DCs) capture and process antigens with high efficiency. CD4^+^ T cells differentiate toward a Th_1_-polarizing phenotype through the activation of DCs (5). However, A. baumannii has been shown to cause facultative intracellular infection recently, and many studies have confirmed its ability to invade lung epithelial cells and macrophages (1, 6–8). Therefore, many immune cells and cytokines that act against intracellular and extracellular infection might be involved in the immune response against *A. baumannii* infection, which is worth of further study. There have been few clinical studies on the changes and specific roles of lymphocyte subsets in the pathogenesis of ARDS caused by *A. baumannii* pneumonia. In this study we aimed to explore the role of lymphocyte subsets in ARDS caused by *A. baumannii* and its correlation with prognosis.

## Methods

We screened all the ARDS patients according to the 2012 Berlin definition (9) admitted to the intensive care unit (ICU) of Peking Union Medical College Hospital (PUMCH) between January 1, 2017 and May 30, 2020. Pulmonary ARDS caused by *A. baumannii* pneumonia and required mechanical ventilation or vasopressors were enrolled in our study. All eligible patients needed to be over 18 years old, ICU stays for 48 h and met none of the exclusion criteria. Exclusion criteria were: (1) any condition causing neutropenia as receiving corticosteroids or immunosuppression; (2) pregnancy or lactation; (3) any condition causing primary or acquired immunodeficiency, such as HIV infection, active autoimmune disease, hematopathy, or malignant tumors receiving chemotherapy within the previous 3 months; and (4) life expectancy <48 h. This study was approved by the Institutional Review Board of PUMCH (approval number: JS-1170), and all methods were performed in accordance with the relevant guidelines and regulations. Informed consent was obtained from all patients, and the study was registered at chictr.org.cn (identifier ChiCTR-ROC-17010750).

The Berlin definition of ARDS included 4 aspects (9): (1) Timing: newly onset or worsening respiratory syndrome within 1 week of known clinical insults; (2) Chest imaging: Bilateral opacities—not fully explained by effusion, lobar/lung collapse, or nodules; (3) Origin of edeme: respiratory failure that can not be fully explained by cardiac failure or fluid overload and need objective assessment to exclude hydrostatic edema if no risk factors present, such as echocardiography; (4) Oxygenation: Mild ARDS 200 mm Hg < PaO_2_/FIO_2_ ≤300 mm Hg with PEEP or CPAP ≥5 cm H_2_O, Moderate ARDS 100 mm Hg < PaO_2_/FIO_2_ ≤200 mm Hg with PEEP≥5 cm H_2_O, Severe ARDS PaO_2_/FIO_2_ ≤100 mm Hg with PEEP ≥5 cm H_2_O.

Clinical diagnostic criteria of pneumonia (10–12): Pneumonia was diagnosed pulmonary infiltrates caused by infection, and at least two of the following findings: fever with a body temperature >38°C or hypothermia with a temperature <36°C; leukocytosis (>12,000 cells/mm^3^) or leukopenia (<4,000 cells/mm^3^); presence of newly purulent tracheal secretions; and a decrease in oxygenation.

Microbiological methods to diagnose pneumonia (12): lower respiratory tract specimens were obtained immediately after ICU admission and were sent to the PUMCH Clinical Microbiology Laboratory. Samples were obtained using non-invasive sampling and cultured semi-quantitatively. Endotracheal aspirates with >25 neutrophils on Gram's stain with <10 epithelial cells per high-power field were required for culture. If qualified samples of suputum were difficult to obtain or diagnose pneumonia, invasive respiratory sampling with bronchoalveolar lavage, protected specimen brush and blind bronchial sampling could be used. The Clinical Microbiology Laboratory determined antimicrobial susceptibility of isolated bacteria by means of the microdilution method (MicroScan System; Baxter health Care, West Sacramento, CA, USA). Results were interpreted according to breakpoints defined by the National Committee for Clinical Laboratory Standards (13). *A. baumannii* had to be the only pathogenic bacterium isolated from the enrolled patients. Bacterial colonization and ventilator-associated tracheobronchitis was carefully excluded by more than two intensivists, and other infections were cautiously excluded.

After enrollment, the following baseline information was collected: age, sex, comorbidities, treatment strategies, ventilator parameters, antibiotics, lymphocyte subsets, cytokines, and other laboratory data. SOFA score, Acute Physiology and Chronic Health Evaluation II (APACHE II) score and Clinical Pulmonary Infection Score (CPIS) within 24 h of admission were calculated, as well as the duration of mechanical ventilation, ICU stay and hospital stay, and 28-day mortality.

Lymphocyte subsets were evaluated in the Infection Laboratory. Peripheral blood mononuclear cells were stained with fluorescent monoclonal antibodies, then subjected to flow cytometric analysis (3-Color EPICS-XL Flow Cytometer; Beckman Coulter, Brea, CA, USA) to detect T cells (CD3^+^), CD4^+^ T cell subgroups, CD8^+^ T cell subgroups, B cells (CD19^+^), and NK cells (CD3^+^CD16^+^CD56^+^). Rate nephelometry (Array 360; Beckman Coulter) was used to measure serum levels of IgA, IgG, and IgM and complement factors C3 and C4.

Initial and targeted antibiotics: initial antibiotics were those given empirically at admission. Targeted antibiotics were those sensitive to *A. baumannii* in *in vitro* sensitivity tests, commonly including ampicillin/sulbactam, cefoperazone sulbactam, amikacin, ceftazidime averbatan, minocycline, carbapenems, tigecycline, and polymyxins.

The study protocol did not call for a standardized approach to critical care, and all treatment measures and drug selections were decided by the intensivists. As circulatory failure happened in most of patients, ECMO was not commonly used in this group.

### Statistical Analysis

Normally distributed data were expressed as the mean and standard deviation and were compared using Students' *t*-test. Non-normally distributed data were presented as median and interquartile range (IQR) and were analyzed using the non-parametric Mann–Whitney *U*-test. Categorical variables were expressed as number and percentage and were compared with the chi-square or Fisher's exact test. A Cox proportional hazards model were performed successively to determine the association between lymphocyte subsets and outcome. Receiver operating characteristic (ROC) curve analysis was performed to determine the discriminatory ability of parameters for predicting 28-day mortality. Youden's index was defined for points along the ROC curve, and the reliability was assessed by sensitivity and specificity. *P* < 0.05 was considered statistically significant. The results were expressed as *P*-value and hazard ratio (OR) with 95% confidence interval (CI). IBM SPSS 23.0 software was used for all statistical analyses (IBM Corp., Armonk, NY, USA).

## Results

**Figure 1:**
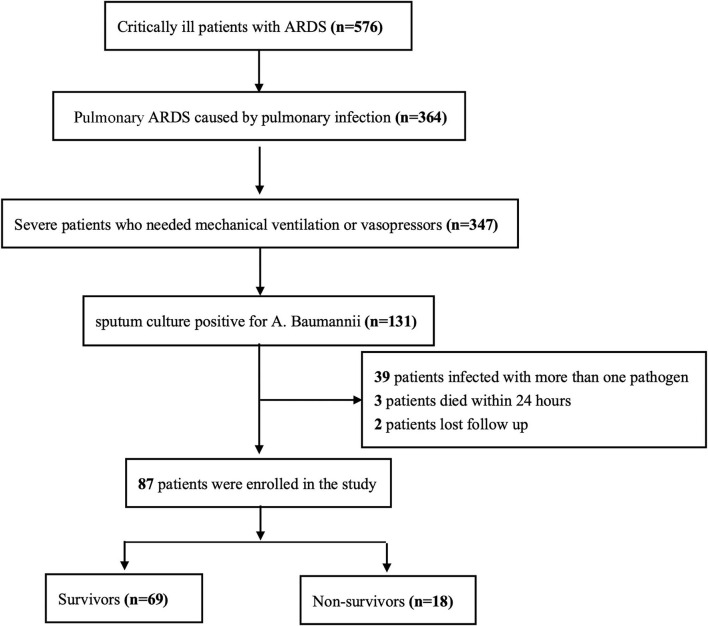
Enrollment flowchart. *A. baumanni, Acinetobacter baumannii*.

A total of 576 ARDS patients based on the Berlin definition were admitted to our ICU during the study period. There were 364 ARDS patients caused by pulmonary infection; 347 of which were severe enough to receive mechanical ventilation or vasopressor treatment. Cultures from the lower respiratory tract of 131 patients were positive for *A. baumannii*. Among the 131 patients with pulmonary ARDS, 39 were infected with more than one pathogen, three died within 24 h after admission, and two were lost to follow-up. Finally, 87 pulmonary ARDS patients caused by *A. baumannii* pneumonia were enrolled in the study (Figure 1).

### Baseline Characteristics of Pulmonary ARDS Patients Caused by *A. baumannii*

The median age of these ARDS patients caused by *A. baumannii* was 66 years (IQR 57–71 years), with males accounting for 70.1% (61/87). The 28-day mortality of these patients was 20.7% (18/87), and there were no significant differences in sex, age, and comorbidities between survivors and non-survivors. According to the 28-day mortality, the severity of the disease was slightly higher in non-survival group than that of survival group, as the differences in SOFA core, APACHE II score, and CPIS score at admission were not significant. The ICU and hospital stays were significantly longer in the survivors (20 vs. 10 days, *P* = 0.004, and 21 vs. 11.5 days, *P* = 0.005, respectively) (Table 1).

**Table 1:** Baseline characters of ARDS patients caused by *A. baumannii* pneumonia.

	**All (***n*** = 87)**	**Survivors (***n*** = 69)**	**Non-survivors (***n*** = 18)**	***P*** **-value**
**Baseline characteristics**				
Sex (male%)	61 (70.1%)	51 (73.9%)	10 (55.6%)	0.13
Age (years) (M, IQR)	66 (57, 71)	66 (57, 71)	64.5 (58, 73)	0.846
**Comorbidities**				
Chronic pulmonary disease	3 (3.4%)	2 (2.9%)	1 (5.6%)	0.582
Diabetes mellitus	33 (37.9%)	23 (33.3%)	10 (55.6%)	0.084
Cardiovascular disease	45 (51.7%)	38 (55.1%)	7 (38.9%)	0.241
Chronic kidney disease	6 (6.9%)	5 (7.2%)	1 (5.6%)	0.801
Autoimmune disease	4 (4.6%)	3 (4.3%)	1 (5.6%)	0.828
Hepatopathy	2 (2.3%)	2 (2.9%)	0	0.465
Malignant tumor	10 (11.5%)	8 (11.6%)	2 (11.1%)	0.954
SOFA score	11.5 (9.75, 14)	11 (9, 14)	12 (10, 15.5)	0.339
Apache II score	19 (15, 22)	19 (15, 22)	21 (13.5, 26)	0.815
CPIS score	6 (5, 8)	6 (5, 8)	7 (5.5, 9)	0.345
**Prognosis related parameters**				
Ventilation day (days)	6.1 (2.6, 10.6)	7 (2.5, 10.5)	4.2 (3.8, 11)	0.797
ICU stay (days)	16 (10, 27)	20 (11, 29)	10 (4, 17.3)	0.004
Hospital stay (days)	20 (10, 28.5)	21 (12, 32)	11.5 (4, 21.3)	0.005

*ARDS, acute respiratory distress syndrome; A. baumannii, Acinetobacter baumannii; M, Median; IQR, inter quartile range; SOFA, sequential organ failure assessment; Apache II Score, acute physiology and chronic health evaluation II score; CPIS, clinical pulmonary infection score; ICU, intensive care unit*.

*P-value for the comparison of survivors and non-survivors according to 28-day mortality*.

### Clinical Characteristics of ARDS Patients Caused by *A. baumannii* Pneumonia

According to the 28-day mortality, there were no significant differences in vital signs and laboratory results between survivors and non-survivors. In terms of respiratory parameters, the PaO_2_: FiO_2_ (P/F) ratio (198 vs. 178, *P* = 0.173) was lower, the driving pressure (12 vs. 13 cmH_2_O, *P* = 0.083) was higher, and the peak airway pressure (21 vs. 24 cmH_2_O, *P* = 0.016) was significantly higher in the non-survivor group. As for treatment, the non-survivors received more renal replacement therapy and glucocorticoids (23.2 vs. 55.6%, *P* = 0.008 and 11.6 vs. 33.3%, *P* = 0.025, respectively). There was no significant difference between the two groups in the initial empirical antibiotic treatment, including the use of effective antibiotics against *A. baumannii* and the subsequent targeted anti-infective treatment. There was also no significant difference in the time interval from the empirical treatment to the targeted treatment. One patient in each group changed antibiotics because of adverse effects, which had no effect on the final statistical analysis (Table 2).

**Table 2:** Clinical characteristics of ARDS caused by *A. baumannii* pneumonia.

	**All (***N*** = 87)**	**Survivors (***N*** = 69)**	**Non-survivors (***N*** = 18)**	***P***
**Vital signs at admission**				
Temperature (°C)	37.5 ± 0.7	37.6 ± 0.7	37.4 ± 0.9	0.345
Heart rate (per minute)	108 ± 19	107 ± 19	109 ± 21	0.728
MAP (mmHg)	84 ± 13	84 ± 14	86 ± 8	0.453
CVP (mmHg)	9 ± 3	9 ± 3	9 ± 2	0.813
**Laboratory test at admission**				
Platelet (^*^10^9^/L)	160 ± 69	166 ± 69	91 ± 46	0.319
Creatinine (umol/L)	114 ± 56	117 ± 57	102 ± 53	0.323
TBil (umol/L)	23.9 ± 13.2	23.6 ± 13.0	25.4 ± 13.9	0.601
Albumin (g/L)	33 ± 4	33 ± 4	33 ± 4	0.649
cTnI (ug/L)	3.4 ± 5.7	3.3 ± 5.9	3.5 ± 4.7	0.963
Nt-ProBNP (pg/ml)	6658 ± 8615	6944 ± 9036	5372 ± 6625	0.606
PT (seconds)	16.7 ± 4.9	16.6 ± 5.0	16.9 ± 4.8	0.784
APTT-R	1.8 ± 3.8	1.9 ± 4.3	1.4 ± 0.4	0.555
Lactate (mmol/L)	2.0 ± 2.0	1.9 ± 2.1	2.6 ± 1.9	0.205
ScvO2 (%)	71 ± 8.1	71 ± 7.8	71 ± 9.6	0.956
NLR	12.3 (7.4–19.5)	11.8 (7.4–18.5)	17.4 (8.3–25.6)	0.224
**Respiratory parameters at admission**				
Tidal volume (ml)	410 (390–440)	410 (400–440)	400 (380–450)	0.4
PaCO_2_ (mmHg)	39 (34–42)	39 (34.4–42)	38 (31.8–42.3)	0.602
P/F ratio	191 (160–228)	198 (165–229)	178 (155–207)	0.173
PEEP (cmH_2_O)	8 (5–10)	8 (5–10)	8 (6–10)	0.324
Ppeak (cmH_2_O)	22 (18–24)	21 (18–23)	24 (21.5–26.5)	0.016
Driving pressure (cmH_2_O)	12 (10–15)	12 (10–14)	13 (11.5–17.5)	0.083
RR (per minute)	15 (15–18)	15 (15–18)	15 (15–17)	0.642
**Treatment strategies**				
Vasopressor	73(83.9%)	57 (82.6%)	16 (88.9%)	0.518
RRT	26 (29.9%)	16 (23.2%)	10 (55.6%)	0.008
ECMO	2 (2.3%)	2 (2.9%)	0	0.465
Neuroblockade agent	4 (4.6%)	2 (2.9%)	2 (11.1%)	0.138
RM	13 (14.9%)	10 (14.5%)	3 (16.7%)	0.818
Prone position	29 (33.3%)	24 (34.8%)	5 (27.8%)	0.574
GCs	14 (16.1%)	8 (11.6%)	6 (33.3%)	0.025
Immunosuppressor	1 (1.1%)	1 (1.4%)	0	0.607
**Initial empiral antibiotics**				
Antibiotics for GNB	84 (96.6%)	67 (97.1%)	17 (94.4%)	0.582
Effective for A.B.	51 (58.6%)	39 (56.5%)	12 (66.7%)	0.436
Antibiotics for GPB	61 (70.1%)	49 (71%)	12 (66.7%)	0.72
Anti-fungal drugs	30 (34.5%)	21 (30.4%)	9 (50%)	0.12
Antiviral drugs	6 (6.9%)	5 (7.2%)	1 (5.6%)	0.801
Virus co-exist	6 (6.9%)	4 (5.8%)	2 (11.1%)	0.428
Candida co-exist	4 (4.6%)	3 (4.3%)	1 (5.6%)	0.828
**Target antibiotics**				0.671
Tigercycline	56 (64.4%)	43 (62.3%)	13 (72.2%)	
Polymyxins	8 (9.2%)	6 (8.7%)	2 (11.1%)	
Other drugs^*^	19 (21.8%)	17 (24.6%)	2 (11.1%)	
Duration from admission to target antibiotics (hours)	15 (11–22)	16 (12–22)	12.5 (10.8–23.5)	0.463

*ARDS, acute respiratory distress syndrome; A. baumannii (A. B.), Acinetobacter baumannii; MAP, mean arterial blood pressure; CVP, central venous pressure; TBil, total Bilirubin; cTnI, cardiac troponin I; Nt-ProBNP, N-terminal pro brain natriuretic peptide; PT, prothrombin time; APTT-R, activated partial thromboplastin time ratio; ScvO_2_, Central venous oxygen saturation; NLR, neutrophil to lymphocyte ratio; PaCO_2_, arterial partial pressure of carbon dioxide; P/F, ratio PaO_2_/FiO_2_; PEEP, positive end expiratory pressure; Ppeak, peak pressure; RR, respiratory rate; RRT, renal replacement therapy; ECMO, extracorporeal membrane oxygenation; RM, recruitment maneuver; GCs, glucocorticoids; GNB, gram negative bacteria; GPB, gram positive bacteria*.

* *Antibiotics that were effective for AB in vitro besides Tigercycline and Polymyxins, such as Ampicillin sulbactam, Cefoperazone sulbactam, Amikacin, Ceftazidime averbatan, Minocycline, Carbapenems et al*.

*P-value for the comparison of survivors and non-survivors according to 28-day mortality*.

### Comparison of Inflammatory and Immune Parameters in ARDS Caused by *A. baumannii* Pneumonia

According to the 28-day mortality, there was no significant differences in procalcitonin, (1,3)-β-D Glucan, interleukin, Tumor necrosis factor-α and other inflammatory markers, as well as complement and immunoglobulin between the two groups. As for lymphocyte subsets: there was no significant difference in the total number of leukocytes and neutrophils between the two groups. The number of T cells (452 ± 322 vs. 729 ± 364/μl, *P* = 0.004), especially CD8^+^ T cells (104 ± 94 vs. 253 ± 191/μl, *P* = 0.002) was significantly lower in non-survivors than in survivors. The total number of lymphocytes and CD4^+^ T cells was also lower in non-survivors (765 ± 525 vs. 1,016 ± 526/μl, *P* = 0.084 and 332 ± 245 vs. 729 ± 364/μl, *P* = 0.011 respectively) (Table 3).

**Table 3:** Inflammatory and immune related markers of ARDS caused by *A. baumannii* pneumonia.

	**All**	**Survivors (***n =*** 69)**	**Non-survivors (***n =*** 18)**	***P***
**Inflammatory markers**				
PCT (ng/ml)	8.8 ± 13.7	7.8 ± 12.6	12.9 ± 17.1	0.165
BDG (pg/ml)	160 ± 326	155.3 ± 344	180.8 ± 246.8	0.729
GM test (pg/ml)	0.52 ± 0.87	0.55 ± 0.98	0.43 ± 0.21	0.606
hsCRP (mg/L)	106.9 ± 85.8	111.1 ± 86.4	92.8 ± 84.6	0.443
IL-6 (pg/ml)	100.9 ± 179.6	99.6 ± 193.2	105.5 ± 127.7	0.921
IL-8 (pg/ml)	225.4 ± 343.2	249.7 ± 379.7	138.2 ± 129.9	0.324
IL-10 (pg/ml)	17.2 ± 22.8	15.7 ± 23.4	22.3 ± 20.7	0.38
TNF-a (pg/ml)	28.6 ± 37.2	31.5 ± 40.8	16.7 ± 10.9	0.319
**Immune parameters**				
C3 (g/L)	0.80 ± 0.23	0.79 ± 0.23	0.85 ± 0.22	0.307
C4 (g/L)	0.18 ± 0.07	0.17 ± 0.07	0.20 ± 0.09	0.132
IgG (g/L)	10.8 ± 3.9	10.7 ± 3.6	11.1 ± 4.8	0.719
IgA (g/L)	2.6 ± 1.2	2.6 ± 1.3	2.8 ± 0.9	0.429
IgM (g/L)	0.95 ± 0.53	0.92 ± 0.5	1.1 ± 0.6	0.297
**Lymphocyte subsets (cells/ul)**				
White blood cell	13474 ± 8080	13584 ± 8743	13061 ± 5059	0.809
Neutrophil	11769 ± 7665	11659 ± 8170	12194 ± 5475	0.794
Monocyte	580 ± 372	559 ± 349	668 ± 459	0.299
Lymphocyte	963 ± 532	1016 ± 526	765 ± 525	0.084
B lymphocyte	206 ± 250	193 ± 221	252 ± 341	0.375
NK T cell	77 ± 72	84 ± 75	51 ± 49	0.087
T lymphocyte	671 ± 372	729 ± 364	452 ± 322	0.004
CD4^+^ T cell	419 ± 252	443 ± 250	332 ± 245	0.095
CD4^+^CD28^+^ T cell	404 ± 245	427 ± 248	318 ± 221	0.097
CD8^+^ T cell	221 ± 185	253 ± 191	104 ± 94	0.002
CD8^+^CD28^+^ T cell	117 ± 83	132 ± 83	59 ± 58	0.001
Memory CD4^+^ T	292 ± 169	311 ± 162	221 ± 182	0.067
45RA^+^CD4^+^ T	126 ± 114	132 ± 119	103 ± 89	0.348
Naïve CD4^+^ T	118 ± 106	124 ± 111	96 ± 88	0.324
CD8^+^DR^+^ T	131 ± 161	151 ± 174	58 ± 61.9	0.029
CD8^+^CD38^+^ T	128 ± 159	151 ± 172	44 ± 36	0.011
CD4^+^ T/CD8^+^ T	2.9 ± 1.4	2.5 ± 2.2	4.0 ± 2.8	0.018

*ARDS, acute respiratory distress syndrome; A. baumannii, Acinetobacter baumannii; PCT, procalcitonin; BDG, (1,3)-β-D Glucan; GM, test Galactomannan test; hsCRP, hypersensitive C-reactive protein; IL, interleukin; TNF-a, tumor necrosis factor-a; C3, complement 3; Ig, immunoglobulin; NK T cell, natural killer T cell*.

*P-value for the comparison of survivors and non-survivors according to 28-day mortality*.

### Parameters Associated With 28-Day Mortality

To clarify the factors independently associated with 28-day mortality, A cox proportional hazards model were performed. SOFA score, lactate level, P/F ratio and driving pressure that had been confirmed to be related to survival were included in the model as well as the parameters that were significantly different between the survivors and non-survivors in our study (Ppeak, RRT and Glucocorticoids use). Lymphocyte related parameters were also included in the model separately. CD8^+^ T cell count and its subtypes were independent factors associated with 28-day mortality of ARDS caused by *A. baumannii* pneumonia (CD8^+^ T cell count: OR 3.667, 95% CI 1.553–8.659, *P* = 0.003; CD8^+^CD28^+^ T cell count: OR 2.043, 95% CI 1.871–31.801, *P* = 0.005; CD8^+^CD38^+^ T cell count: OR 2.705, 95% CI 1.152–6.353, *P* = 0.022) (Table 4).

**Table 4:** Cox regression of lymphocyte subsets related parameters and survival.

	**B**	**SE**	**Wald**	**OR**	**95%CI**	***P*** **-value**
CD8^+^ T cell count (cells/ul)	1.3	0.438	8.789	3.667	1.553, 8.659	0.003
CD8^+^CD28^+^ T count (cells/ul)	2.043	0.723	7.992	7.714	1.871, 31.801	0.005
CD8^+^CD38^+^ T count (cells/ul)	0.995	0.436	5.222	2.705	1.152, 6.353	0.022

*SE, standard error of the mean; OR, odds ratio; 95% CI, 95% confidence interval*.

**Figure 2:**
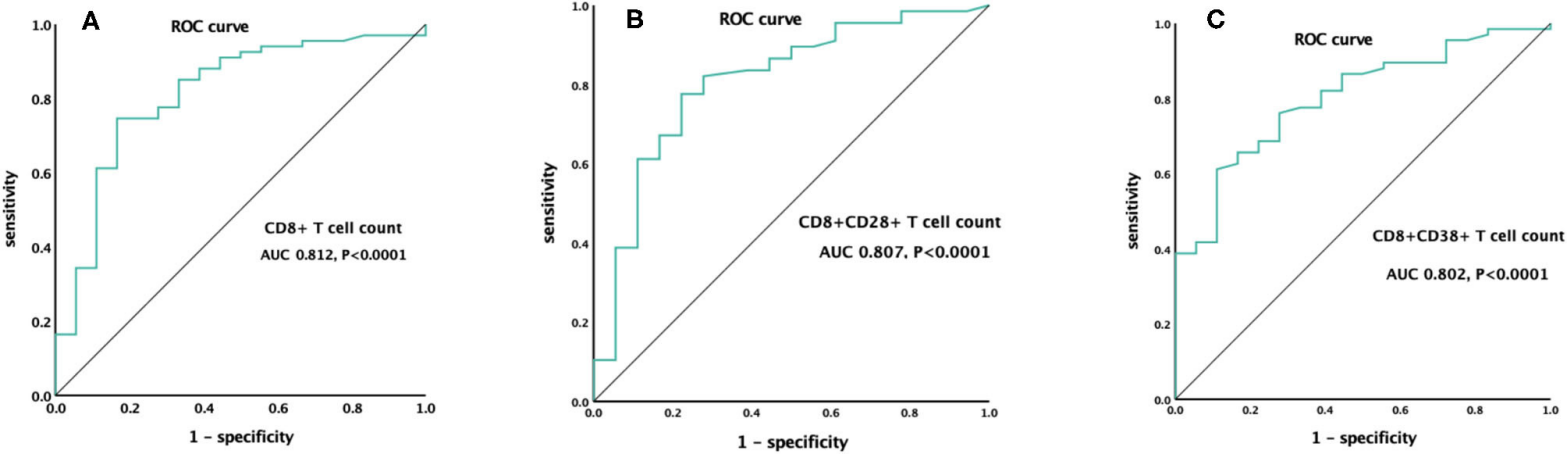
ROC curve of CD8^+^ T cell count **(A)**, CD8^+^CD28^+^ T cell count **(B)**, and CD8^+^CD38^+^ T cell count **(C)** for the prediction of 28-day mortality.

To further analyze the influence of lymphocyte subsets related parameters that differed significantly between survivors and non-survivors in the univariate analysis on prognosis, ROC curve analysis was performed. The CD8^+^ T cell count had the greatest discriminatory ability, with an area under the curve of 0.812. A cutoff value of 123 cells/μl at ICU admission was predictive of 28-day mortality for ARDS caused by *A. baumannii* with a sensitivity of 74.6% and specificity of 83.3% (*P* < 0.0001) (Figure 2).

**Figure 3:**
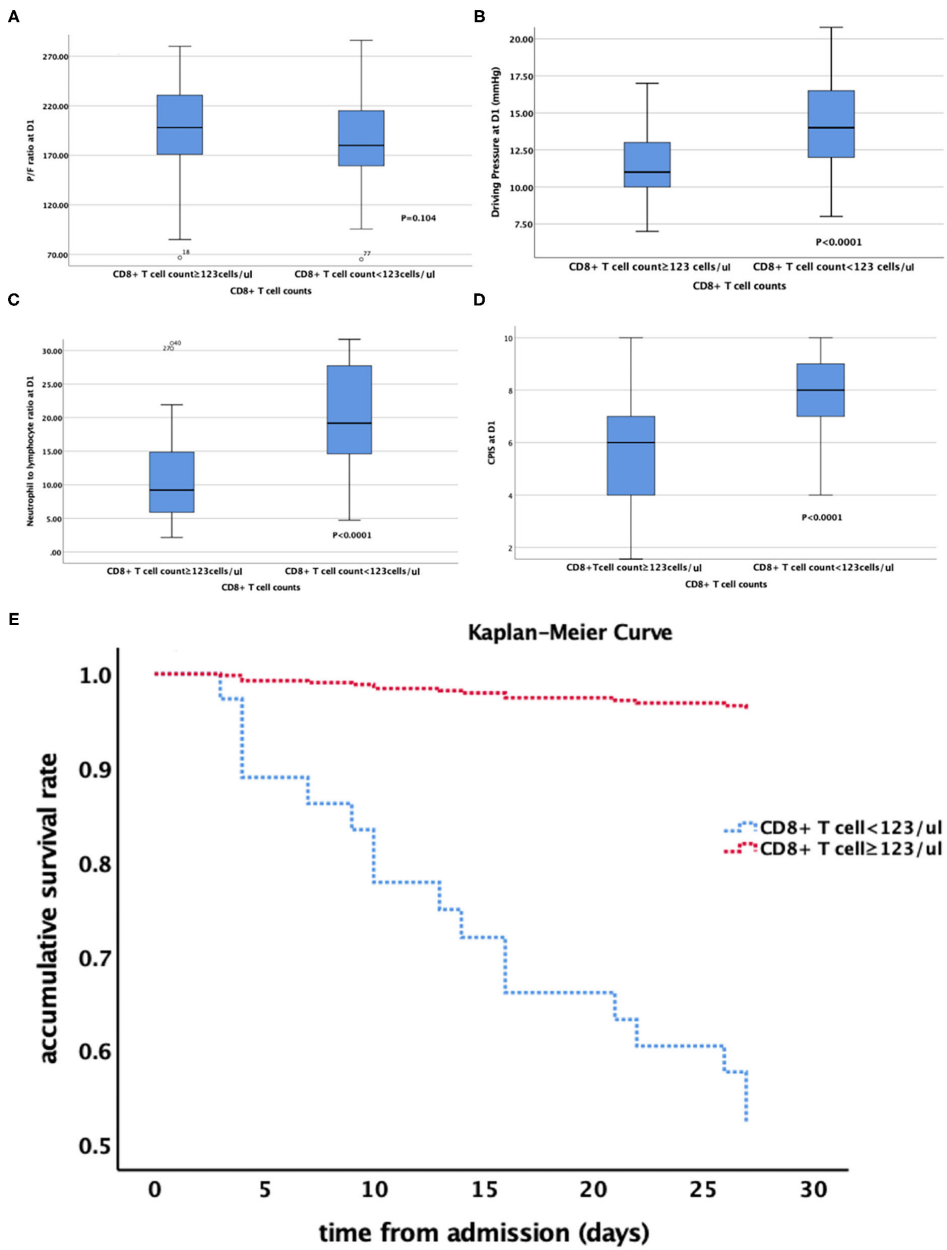
**(A–E)** relationship between CD8^+^ T cell count and **(A)** P/F ratio; **(B)** Driving Pressure; **(C)** Neutrophil to lymphocyte ratio; **(D)** CPIS. **(E)** the Kaplan-Meier curve of patients with different CD8^+^ T cell counts. P/F ratio at D1 PaO_2_:FiO_2_ at admission; CPIS, clinical pulmonary infection score.

**Figure 4:**
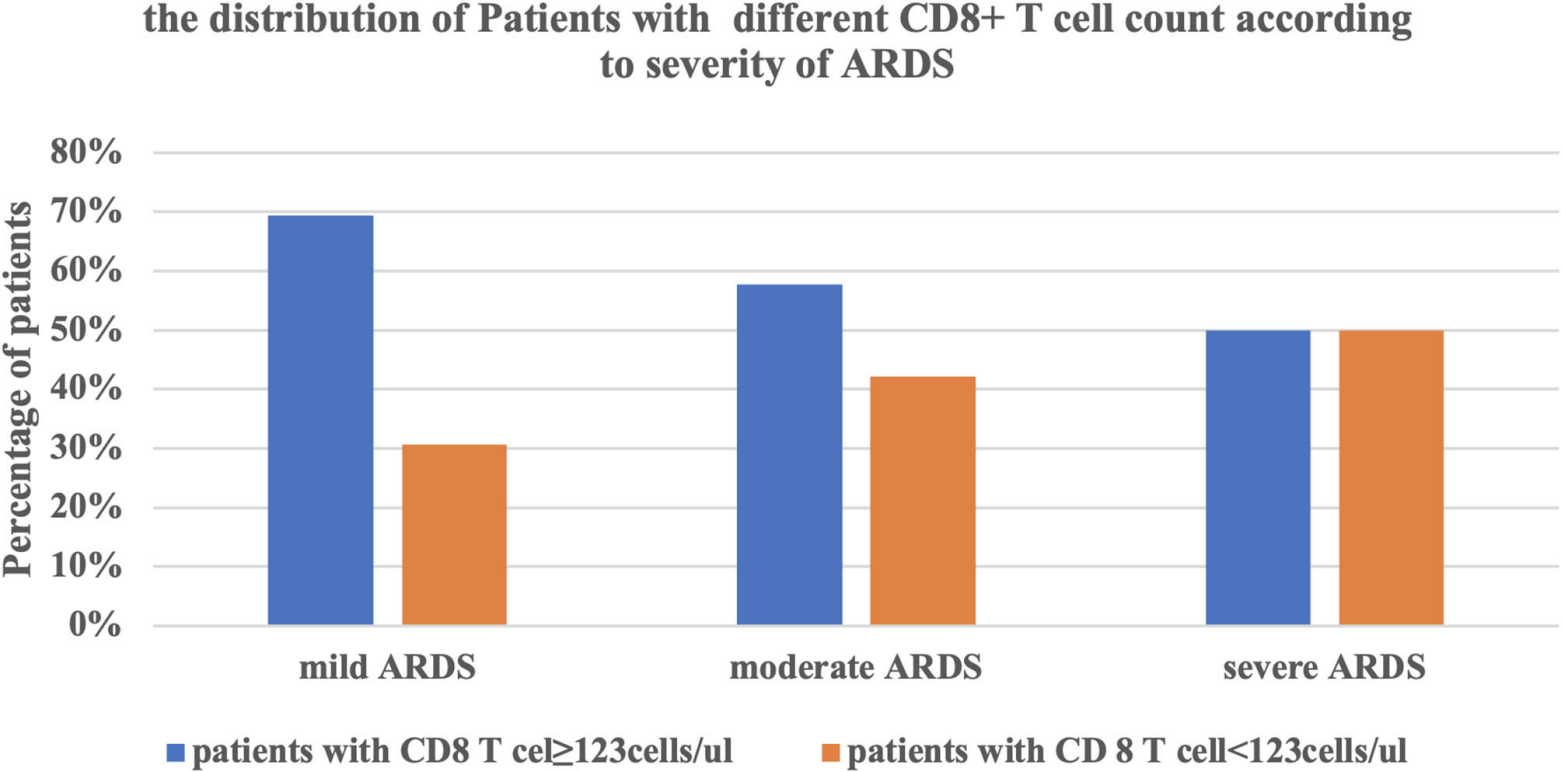
The relationship between the severity of ARDS and the CD8^+^ T cell counts. ARDS, acute respiratory distress syndrome.

Furthermore, we divided these ARDS patients into two groups according to the CD8^+^ T cell count (<123 and ≥123 cells/μl). the accumulative survival rate from the Kaplan-Meier curve was significantly lower and ventilation free day was significantly shorter (14.5 vs. 21 days, *P* = 0.001) in patients with a lower CD8^+^ T cell count, while 28-day mortality was significantly higher than that in patients with a higher CD8^+^ T cell count (46.9 vs. 5.7%, *P* < 0.0001). The patients with lower CD8^+^ T cell counts had lower P/F ratio, higher driving pressure at admission. The NLR, PCT level and CPIS at admission were also higher in patients with a lower CD8^+^ T cell count (Table 5, Figures 3A–E). We divided the cohort into different severity of ARDS according to admission P/F ratio and found that patients with increasing severity of ARDS had progressively lower CD8^+^ T cell counts (Figure 4).

**Table 5:** Comparison of ARDS patients with different CD8^+^ T cell counts.

	**CD8^+^ T cell ≥123 (***n =*** 53)**	**CD8^+^ T cell <123 (***n =*** 32)**	***P***
**Respiratory parameters of D1**			
Tidal volume (ml)	410 (392–448)	400 (380–440)	0.428
PEEP (cmH_2_O)	8 (5–9.5)	8 (6–10)	0.222
Ppeak (cmH_2_O)	20.5 (18–23)	23 (20–25)	0.002
Driving pressure (cmH_2_O)	11 (10–13)	14 (12–17)	<0.0001
RR (per minute)	21 (18–23)	23 (20–25)	0.018
P/F ratio	198 (171–235)	178 (157–212)	0.104
PaCO_2_ (mmHg)	38 (34–42)	39 (33–42)	0.978
**Inflammatory markers of D1**			
NLR	10.1 (5.9–15.1)	19.2 (12.5–29.1)	<0.0001
PCT (ng/ml)	5.8 ± 11.3	12.8 ± 15.5	0.022
IL-6 (pg/ml)	120.4 ± 219.7	72.3 ± 96.1	0.35
IL-8 (pg/ml)	211.4 ± 317.2	234.3 ± 391	0.814
IL-10 (pg/ml)	18.3 ± 26.8	15.7 ± 15.6	0.692
TNF-a (pg/ml)	33.4 ± 44.6	19.3 ± 19.9	0.256
**Other parameters**			
CPIS	6 (4–7)	8 (7–9)	<0.0001
SOFA	11 (9–14)	12 (10–14)	0.271
Apache II score	18 (15–22)	20.5 (14–25.3)	0.774
Ventilation day (days)	5.5 (2.5–11)	6.6 (4–10.3)	0.586
28-day ventilation free day (days)	21 (16–25.5)	14.5 (0–20.9)	0.001
28-day Mortality	3 (5.7%)	15 (46.9%)	<0.0001

*ARDS, acute respiratory distress syndrome; D1, the day at admission; D3, 3 days after admission; PEEP, positive end expiratory pressure; Ppeak, peak pressure; RR, respiratory rate; P/F ratio, PaO_2_:FiO_2_; PaCO2 arterial partial pressure of carbon dioxide; NLR, neutrophil to lymphocyte ratio; PCT, procalcitonin; IL, interleukin; TNF-a, tumor necrosis factor-a; CPIS, clinical pulmonary infection score; SOFA, sequential organ failure assessment; Apache II, Score acute physiology and chronic health evaluation II score*.

*P-value for the comparison of patients with higher (≥123cells/ul) and lower (<123cells/ul) CD8^+^ T cell count*.

## Discussion

To our knowledge, this is the first clinical study exploring the early alterations of lymphocyte subsets in ARDS caused by *A. baumannii* pneumonia. 28-day mortality of these patients was 20.7%. The T cell count, especially for CD8^+^ T cells, at admission was independently associated with 28-day mortality. The CD8^+^ T cell count was an early predictive marker for prognosis. So far, studies about *A. baumannii* and immunity have been limited to basic research, and there are limited data for clinical use. Our study first confirmed that T cell immunity might play an important role in ARDS caused by *A. baumannii* pneumonia, which could be important for clinical practice.

Immune disorder is the characteristic manifestation of sepsis, and increasing evidence shows that the immune system plays a bridging role between severe infection, organ damage and prognosis. However, the current view of how the two most complex syndromes, systemic inflammatory response syndrome and immune response syndrome, affect each other is still rudimentary (14, 15). The first step is to clarify the relationship between certain diseases and immune responses. There is a profound relationship between NLR and sepsis, NLR and ARDS, the CD4^+^ T cell count and carbapenem-resistant Enterobacteriaceae (CRE) infection (16), and there has been growing interest in identifying biological sub-phenotypes of ARDS patients with similar host-response features for prognostic enrichment (3). Basic immunological research into *A. baumannii* infection is in full swing, along with research into the mechanism of host defense against the infection. For example, there has been in-depth study of outer membrane protein A (5, 17). However, there has been little clinical research on the relationship between immunity to *A. baumannii* infection and prognosis. In this study, we found that the CD8^+^ and CD8^+^CD28^+^ T cell counts were independently associated with 28-day mortality in ARDS patients caused by *A. baumannii*. Clinically, this is the first-time providing evidence of Acinetobacter baumannii causing intracellular infection, which makes the understanding of this aspect go further.

A variety of immune cells are involved in resistance to ARDS caused by *A. baumannii* pneumonia (18). Large numbers of *A. baumannii* are taken up by alveolar macrophages as early as 4 h after infection. NK T cells are another cell type that act during the immune response against *A. baumannii*. Depletion of NK T cells in a murine pneumonia model caused impaired bacterial clearance and increased mortality. We found that the NK T cell count in non-survivors was lower than in survivors, which was consistent with previous research (19). DCs are the bridge between innate and adaptive immune responses, CD4^+^ T cells differentiate toward a Th_1_-polarizing phenotype through the activation of DCs (5). Subsequently, CD4^+^ T-helper cells support the production of specific antibodies by B cells and promote the bactericidal activity of phagocytes that together clear the infection. Our previous study also illustrated that the CD4^+^CD28^+^ T cell count was a useful marker for early diagnosis of CRE infection and outcome prediction (16). It was not surprising to see that innate immune cells were involved in the defense against *A. baumannii*, but with the ability to cause intracellular infection, *A. baumannii* pneumonia should induce cellular immunity response. However, the relationship between the CD8^+^ T cell count and *A. baumannii* has rarely been studied before (6). For the first time, our study illustrated the association between CD8^+^ T cells and ARDS caused by *A. baumannii* pneumonia. The possible mechanisms were as follows: (1) *A. baumannii* directly invaded alveolar epithelial cells, and cytotoxic CD8^+^ T cells directly recognized and killed infected epithelial cells (8); and (2) in addition to CD8^+^ T cells, the CD8^+^CD38^+^ T cells also differed significantly between survivors and non-survivors with *A. baumannii* infection. Inflammatory reaction, antigen exposure and environmental factors have been shown to allow differentiation of the NK-like CD8^+^ T cells. Therefore, CD8^+^ cells may also act indirectly against *A. baumannii* infection, which could be important for improving protective immunity (20). Further laboratory and clinical studies are needed to explore the specific mechanism of CD8^+^ T cells in *A. baumannii* infection.

Our study showed that the CD8^+^ T cell count was an independent risk factor for 28-day mortality of ARDS caused by *A. baumannii* pneumonia, which could be explained by the difference in physical and biological sub-phenotypes of ARDS patients (3). From the perspective of biological phenotype, multiple large clinical ARDS trials used P/F ratio and driving pressure for prognostic enrichment. The CD8^+^ T cell count was significantly associated with P/F ratio in ARDS caused by *A. baumannii* pneumonia and stratified according to P/F ratio. The proportion of patients with a lower CD8^+^ T cell count was significantly higher in severe ARDS patients. The CD8^+^ T cell count was also significantly associated with driving pressure, and patients with a lower T cell count needed higher driving pressure. Both severity of ARDS and driving pressure were strongly associated with survival rate (2, 21). From the perspective of biological phenotype, this might be related to the intensity of local inflammatory response. T cells, especially CD8^+^ T cells, were significantly decreased in non-survivors, resulting in a significant increase in NLR. The adaptive immune response to *A. baumannii* was reduced, while the non-specific innate immunity mainly composed of neutrophils was enhanced, which led to enhancement of the local pro-inflammatory response, manifesting as higher CPIS.

There were several limitations to this study. First, the study lasted almost 3 years with extremely strict inclusion criteria. Only septic patients with ARDS whose only pathogen was *A. baumannii* were enrolled, and they needed to receive mechanical ventilation or vasopressor treatment for enrollment. Despite the relatively small sample size, it was large enough to illustrate the role of lymphocytes, especially CD8^+^ T cells in patients with ARDS caused by *A. baumannii*. Second, the serum lymphocyte count might not be able to reflect the local infection situation. We need to further study the cell subgroup analysis of bronchoalveolar lavage fluid (BALF). However, there are challenges because, at present, the cell classification of BALF cannot be quantified as in routine blood tests, and there is no normal reference range for the cell classification; both of which need further research. Last, one key element that stands out from the existing studies is how much strain-to-strain variation of *A. baumannii* influences the interaction with host cells and observed phenotypes *in vivo*. A deeper understanding of the virulence factors of *A. baumannii* that are important for *in vivo* pathogenesis is needed (1).

## Conclusions

Lower CD8+ T cell count was associated with higher severity and early mortality in ARDS patients caused by A. *baumannii* pneumonia, which could be valuable for outcome prediction.

## Data Availability Statement

The raw data supporting the conclusions of this article will be made available by the authors, without undue reservation.

## Ethics Statement

The studies involving human participants were reviewed and approved by the Institutional Review Board of PUMCH (approval number: JS-1170). The patients/participants provided their written informed consent to participate in this study.

## Author Contributions

WC and NC contributed to the conception of the study, data interpretation, and drafted the manuscript. HW, JZ, DL, GB, WH, and JC contributed to data collection and critically revised the manuscript for important intellectual content. All authors approved the final version of the manuscript.

## Funding

This work was supported by National Natural Science Foundation of China (Nos. 82072226 and 81601657), Beijing Municipal Science and Technology Commission (No. Z201100005520049), Non-profit Central Research Institute Fund of Chinese Academy of Medical Sciences (No. 2019XK320040), Tibet Natural Science Foundation [No. XZ2019ZR-ZY12(Z)], and Excellence Program of Key Clinical Specialty of Beijing in 2020 (No. ZK128001).

## Conflict of Interest

The authors declare that the research was conducted in the absence of any commercial or financial relationships that could be construed as a potential conflict of interest.

## Publisher's Note

All claims expressed in this article are solely those of the authors and do not necessarily represent those of their affiliated organizations, or those of the publisher, the editors and the reviewers. Any product that may be evaluated in this article, or claim that may be made by its manufacturer, is not guaranteed or endorsed by the publisher.

## Abbreviations

ARDS: acute respiratory distress syndrome
*A. baumannii*: Acinetobacter *baumannii*
ICU: intensive care unit
NLR: neutrophil to lymphocyte ratio
TNF: tumor necrosis factor
DCs: dendritic cells
NK: natural killer cells
SOFA: sequential organ failure assessment score
CPIS: clinical pulmonary infection score
Ig: immunoglobulin
IQR: interquartile range
ROC: Receiver operating characteristic curve
OR: odds ratio
AUC: area under the curve
P/F: ratio PaO_a_: FiO_2_
BALF: bronchoalveolar lavage fluid.
